# The Mersin Greenhouse Workers Study. Surveillance of Work-related Skin, Respiratory, and Musculoskeletal Diseases

**DOI:** 10.29024/aogh.2315

**Published:** 2018-08-31

**Authors:** Aydın Nuraydın, Özgür Bilek, Ali Koray Kenziman, Mehmet Ali Korkusuz, Ali İhsan Atagün, Nezaket Özpolat Çakar, Naci Özer, Serdar Deniz, Mustafa Kemal Başaralı, Ahmet Özlu, Abdulsamet Sandal, Gert van der Laan, Ali Naci Yıldız

**Affiliations:** 1 Ministry of Health, Mersin Public Health Directorate, Mersin, TR; 2 Ministry of Health, Mardin Public Health Directorate, Mardin, TR; 3 Ministry of Health, Manisa Public Health Directorate, Manisa, TR; 4 Ministry of Health, Turkish Public Health Institution, Ankara, TR; 5 Hacettepe University, Faculty of Medicine, Department of Internal Medicine, Division of Occupational Medicine, Ankara, TR; 6 Department of Health Sciences of the University of Milan, Milan, IT; 7 Medical Centre of Vrije Universiteit Amsterdam, Amsterdam, NL; 8 Hacettepe University, Faculty of Medicine, Department of Public Health, Ankara, TR

## Abstract

**Background:**

Skin, respiratory, and musculoskeletal diseases in greenhouse workers are frequently observed due to exposure to plant products and pesticides in enclosed conditions and ergonomic risks. Current studies on occupational health risks of greenhouse workers in Turkey are insufficient.

**Objectives:**

The aim of the present study was to assess work-related skin, respiratory, and musculoskeletal diseases in greenhouse workers in the Erdemli province, Mersin, an area with a workforce predominantly active as greenhouse workers.

**Methods:**

The study population consists of adult greenhouse workers, who visited their family physician between June 12–14, 2017 and were diagnosed with dermatological, respiratory, and musculoskeletal diseases. Immediately after this consultation, occupational physicians conducted face-to-face interviews, asking questions about sociodemographic features and occupational factors including current and previous work, current exposures and relation of current symptoms with work.

**Results:**

In total, 423 workers with 555 diagnoses were included in the study. Percentages of diagnoses were 30.1%, 21.6% and 48.3%, for skin, respiratory and musculoskeletal diseases respectively. Nearly half of participants had taken an absence from work due to those diseases. Mean age of onset for greenhouse working was 15.5 years. Almost all participants (96.2%) reported contact with chemicals, and usage of respiratory protection was low (17.3%). Pesticides were regarded as a risk factor by nearly two-thirds of workers with skin or respiratory system disorders. Participants’ answers to questions regarding the relationship between their diseases and their work was positive for more than half of patients and patients with skin and respiratory diseases. Nearly half of the patients with musculoskeletal diseases attributed their complaints to physical overload at work.

**Conclusions:**

We found evidence of work-relatedness in almost half of the 555 greenhouse workers with dermatological, respiratory and musculoskeletal diseases. These findings are helpful in creating an awareness program. There is a need for a more detailed assessment of the cases and the related working conditions to start a tailored prevention program.

## Introduction

The share of the population working in agriculture greatly varies among countries: while the vast majority of the population in low-income countries work in agriculture, this rate drops to less than 5% in high-income countries [1]. In Turkey, agriculture employs nearly one-fifth (18.9%) of the registered workforce [2]. The work is generally heavy, working hours can be very long, workers are exposed to extreme climatic conditions, and many are also exposed to hazardous chemicals, particularly pesticides [3]. Agriculture and forestry, as an occupation, consistently ranks as the third or fourth most hazardous occupations in the European Union [4]. Agricultural workers suffer from several conditions and diseases [5]. Farmers and farm workers experience high rates of low back, shoulder, and upper extremity disorders [6]. Pesticide-related illnesses refer to a broad group of health outcomes, including dermatitis, eye injuries, respiratory diseases, and cancers [7]. The use of pesticides causes at least 7 million cases of acute and long-term nonfatal illness [8].

Greenhouse cultivation, a form of agricultural production, is commonly preferred among farmers because it allows farmers to grow vegetables, fruit, flowers, rare and exotic plants outside the normal seasons in areas covered by glass or plastic, regardless of weather conditions. The work has many physical, chemical, biological and ergonomic risks, and those risks result in a variety of occupational accidents and work-related or occupational diseases [4,9]. Although climate conditions can be controlled in greenhouse cultivation, the level of occupational exposures to pesticides and plant products and related health risks in greenhouse workers are higher than those of outdoor farm workers due to the enclosed working conditions. The main hazards of working in greenhouses include: injury from materials, problems related to working position, chemicals including pesticides, endotoxins, dusts, and work at height [4,10]. Dermatitis, musculoskeletal complaints, respiratory system diseases, and neurological diseases are the most common diseases among greenhouse workers [11]. However, the number of studies on health and safety issues of greenhouse workers is limited.

In Turkey, there are approximately 5 million agricultural workers, but there was only one recognized case of occupational disease in the agricultural work sector between 2012 and 2015, according to official statistics of the Social Security Institution (SSI) [2,12]. There are no official statistics of occupational diseases in greenhouse workers. Greenhouse agriculture is common in the southern Turkey where Mersin is located. The Erdemli province of Mersin is one of the leading regions of Turkey’s greenhouse production. The main economic activity in the district is agriculture, for which greenhouse cultivation is an important form of production. Enterprises are mostly small-scale family enterprises. The main products are tomato, cucumber, pepper, and banana. Despite the extent of this economic activity in this region, there is no data about the frequency occupational diseases of greenhouse workers. This gap in the literature was recently noticed by the occupational physicians in the region who are also part of the current research team. Thus, in this study, we aimed to assess the greenhouse workers’ perception and evaluation of occupational risk factors for dermatological, respiratory, and musculoskeletal diseases.

## Materials and Methods

The research was conducted between June 12–July 14, 2017 (during 23 work days) in three family health centers (FHCs), which offer health service to a region with numerous greenhouses in the Erdemli province. Nearly 21,000 people are served by nine family physicians working in those three FHCs. Greenhouse workers over 18 years of age who were diagnosed with dermatological, respiratory, and musculoskeletal diseases by family physicians within the study period and who agreed to participate to the study were included. After the family physician visit, patients meeting specified criteria were asked whether they wanted to participate in the ongoing research. Patients who accepted were directed to one of the six occupational physicians for data collection via face-to-face interview in FHCs. Data collection forms had 23 questions divided into in two parts, including sociodemographic features and features related to work including current and previous work, current exposures, and relation of current diagnosis with work. Diagnoses obtained from the family physician visit were listed according to 10th edition of the International Statistical Classification of Diseases and Related Health Problems (ICD-10) codes [13], but no additional examinations were performed due to limited resources, nor was any treatment recommended by the researchers. The preliminary study was performed on ten patients from a different FHC.

Researchers obtained informed consent from each of the participants, whose names were not recorded. After the interviews, all participants were informed about occupational diseases and advised to refer to the related department of the nearest authorized hospital (Mersin University Hospital) to prepare reference files for final diagnosis of occupational diseases by SSI. Permissions were obtained from Ministry of Health Turkish Public Health Institution, Mersin Public Health Directorate and Erdemli District Governorship, and the results of the research were presented to those units. Ethical approval was obtained from Hacettepe University Ethics Committee for Non-Interventional Clinical Studies (30 May 2017, Report No. 2017/14-846).

Statistical analyses were performed using the SPSS software version 24.0. Chi-square test was performed for categorical comparisons and logistic regression analyses were used for multiple comparisons. A 5% type-1 error level was used to infer statistical significance.

## Results

In three FHCs, 487 greenhouse workers over 18 years of age were diagnosed with skin, respiratory or musculoskeletal disease in 23 work days; however, 64 workers did not agree to participate in the study. Some demographic characteristics of 423 participants were summarized in Table 1. More than two-thirds (63.3%) of the participants were between 30–49 years of age and 30 participants (7.1%) were 60 years of age or older. A total of 74.9% of participants started to work in greenhouses at 18 years of age or younger, while 37.5% began such work at 15 years of age or younger.

**Table 1:** Demographic and work characteristics of participants (n = 423).

Characteristic		Statistics

**Age (years)**, mean ± SD (min-median-max)		41.2 ± 11.2 (18–41–75)
**Sex**, n (%)	Male	247 (58.4)
	Female	176 (41.6)

**Business type**, n (%)	Family business	355 (83.9)
	Business owner	28 (6.6)
	Salaried seasonal worker	26 (6.1)
	Paid permanent worker	13 (3.1)

**Age started to work (years)**, mean ± SD (min-median-max)		15.5 ± 3.6 (5–15–35)
**Total working duration in greenhoouses (months)**, mean ± SD (min-median-max)		215.5 ± 116.1 (12–216–732)
**Working duration in current greenhouse (months)**, mean ± SD (min-median-max)		187.5 ± 111.5 (1-180–480)
**Prior working history**, n (%)	Absent	243 (57.4)
	Present*	180 (42.6)

**Duties performed in greenhouses**, n (%)	Dibbling	413 (97.6)
	Weeding	379 (89.6)
	Spraying	364 (86.1)
	Spinning	411 (97.2)
	Fertilization	353 (83.5)
	Harvesting	415 (98.1)
	Sorting	403 (95.3)
	Roofing	323 (76.4)

**Occupational health practice**^†^, n (%)	Contact with chemical substances	405 (96.2)
	Using work clothes	293 (69.6)
	Using gloves	266 (62.9)
	Using work clothes in daily life/at home	192 (45.6)
	Using a mask	73 (17.3)
	Consuming food and beverage in the greenhouse	282 (67)

min, minimum; max, maximum; SD, standard deviation. *Duration of prior working history (years), mean ± SD (min-median-max) was 12.1 ± 7.4 (1–10–41). ^†^Two cases were missing (n = 421).

Of all participants, 57.4% who worked in the current greenhouses had previously worked in another workplace (Table 1); 169 people worked at one other workplace, 11 people worked at two and two participants worked at three different workplaces. Approximately one-fifth of these workplaces (22.8%) were greenhouses. Other sectors (n = 152) were outdoor agriculture and livestock (75.0%), industry (10.5%), service sector (10.5%) and construction sector (3.9%). The mean working duration at previous workplace was 12.1 ± 7.4 years (min = 1, max = 41, median = 10). There were 60 participants (14.2%) working in additional jobs (40.0% service sector, 31.6% outdoor agriculture and livestock, 13.3% greenhouse manufacturing, 8.3% industry, 6.6% construction).

In terms of duties listed in Table 1, three of four participants (75.5%) stated that they performed all of the jobs listed. In the analysis of duties according to sex, statistically significant differences (p < 0.0001) were observed for weeding (63.1%–36.9%), spraying (66.5%–33.5%), fertilization (68.8%–31.2%), roofing (72.1%–27.9%) and workers who did all the jobs listed (72.3%–27.7%) (order of percentages follows as men to women).

Some features of participants related to occupational health practices are given in Table 1. Logistic regression analysis did not reveal any statistically significant relationship between presence of the dermatological or respiratory disease and contact with chemical substances, features related to personal protectors (e.g. usage of special clothes, gloves, and masks at work or changing clothes after work), and consuming food or beverages in greenhouses (p > 0.05).

With 113 people having more than one diagnosis (94 people with two, and 19 people with three systemic diseases), a total of 555 diseases were diagnosed in 423 individuals (Table 2). Durations of the complaints in months [n (mean ± standard deviation-median)] were 167 (80.05 ± 66.19–60) for skin diseases, 120 (112.32 ± 85.0–16) for respiratory diseases, and 266 (92.39 ± 68.90–72) for musculoskeletal diseases.

**Table 2:** Distribution of the diseases according to ICD-10* codes.

Disease		n	%

**Skin (n = 167)**	L23.9 Allergic contact dermatitis, unspecified cause	125	74.9
	L30.9 Dermatitis, unspecified	11	6.6
	L50 Urticaria	9	5.4
	L29.9 Pruritus, unspecified	8	4.8
	L20.9 Atopic dermatitis, unspecified	6	3.6
	R23.8 Other skin changes	5	3.0
	R21 Rash and other nonspecific skin eruption	3	1.8

**Respiratory System (n = 120)**	R06.0 Dyspnea	68	56.7
	J45 Asthma	32	26.7
	J30.4 Allergic rhinitis, unspecified	8	6.7
	R05 Cough	7	5.8
	J40 Bronchitis, not specified as acute or chronic	2	1.7
	R07.0 Pain in throat	2	1.7
	J44 Other chronic obstructive pulmonary disease	1	0.8

**Musculoskeletal System (n=268)**	M54.5 Low back pain	195	72.8
	M25.5 Pain in joint	25	9.3
	M51.9 Unspecified thoracic, thoracolumbar and lumbosacral intervertebral disc disorder	22	8.2
	M79.1 Myalgia	20	7.5
	M79.6 Pain in limb, hand, foot, fingers and toes	4	1.5
	M50 Cervical disc disorders	2	0.7

*10th revision of the International Statistical Classification of Diseases and Related Health Problems.

More than half of the patients with skin or respiratory diseases answered open-ended questions about their own beliefs regarding possible occupational factors causing the disease as pesticides (61.1% and 66.7% respectively). People with musculoskeletal disease most frequently indicated heavy lifting (46.6%) and bending (45.1%) as occupational risk factors (Table 3).

**Table 3:** Distribution of factors participants’ answers about their own beliefs regarding possible occupational factors causing the disease.

Disease		n	%

**Skin (n = 167)**	Pesticides	102	61.1
	Dust	58	34.7
	Hot working environment	27	16.2
	Contact with the produced plant	18	10.8
	Humidity	9	5.4
	Bending work	2	1.2
	Chemical fertilizer	2	1.2
	Working upwards	2	1.2
	Heavy lifting	1	0.6

**Respiratory System (n = 120)**	Pesticides	80	66.7
	Dust	54	45
	Humidity	19	15.8
	Hot working environment	18	15
	Chemical fertilizer	3	2.5
	Heavy lifting	1	0.8

**Musculoskeletal System (n = 268)**	Heavy lifting	125	46.6
	Bending work	121	45.1
	Hot working environment	15	5.6
	Working upwards	8	3
	Dust	2	0.7
	Humidity	2	0.7
	Pesticides	2	0.7

The frequency of any workday loss due to illness in past year was 19.7% (n = 33) for skin diseases, 55.8% (n = 67) for respiratory diseases, 57.5% (n = 153) for musculoskeletal diseases, and 45.7% (n = 253) overall. The frequency of workday loss from respiratory and musculoskeletal disorders was significantly higher than that from skin disease (p < 0.001).

Analysis of answers of participants to questions asking the relationship between their complaints or diseases and their work revealed that more than half of the all patients and patients with skin diseases or patients with musculoskeletal diseases answered positive to all five questions (50.1%, 50.9%, and 57.1% respectively). Only five people (1.2%) answered negatively to all questions (Table 4). In the logistic regression analysis, a positive response to the occupational relationship for all five problems involving the work performed by the patient was not associated with sex, age, previous job, previous occupation, other job, current disease duration, the number of days in which the worker did not work in the last year and the working position (p > 0.05). Compared with the lesser duration of greenhouse work history, those who worked more than 20 years in greenhouses and compared to those diagnosed with a respiratory system disease, those diagnosed with a musculoskeletal system disease were 3.05 times higher (95% CI: 1.55–6.00, p = 0.001) and 2.43 times (95% CI: 1.30–4.50, p = 0.005) answered as yes to all questions respectively.

**Table 4:** Distribution of positive answers for questions asking work-relatedness and for all questions about the relationship of diseases to work.

	Skin (n* = 167)		Respiratory System (n* = 120)		Musculoskeletal System (n* = 268)		Total (n^†^ = 423)	

Question	n	%	n	%	n	%	n	%

Did disease start after started to work in the greenhouse?	141	84.4	97	80.8	230	85.8	353	83.5
Does the disease exacerbated as you work in the greenhouse?	155	92.8	117	97.5	258	96.3	402	95
Are there any others working in the same workplace have similar disease?	102	61.1	56	46.7	186	69.4	262	61.9
Do your complaints decline when you do not work in the greenhouse?	152	91	112	93.3	249	92.9	387	91.5
Is there at least one factor in your workplace causative this disease?	148	88.6	110	91.7	248	92.5	380	89.8
**Total number of positive answers**	n*	**%**	n*	**%**	n*	**%**	**n^†^**	**%**

5	85	50.9	46	38.3	153	57.1	212	50.1
4	54	32.3	53	44.2	84	31.3	144	34,1
3	14	8.4	12	10	17	6.3	36	8.5
2	4	2.4	6	5	7	2.6	14	3.3
1	7	4.2	2	1.7	5	1.9	12	2.8
–	3	1.8	1	0.8	2	0.7	5	1.2

*Number of diagnoses. ^†^Number of workers.

Of the cases referred by family physicians to the Mersin University Hospital, three were related to musculoskeletal diseases, one for respiratory system diseases, and one for skin diseases.

## Discussion

In the present study, we evaluated 423 greenhouse workers with total number of 555 diagnoses of skin, respiratory, and musculoskeletal diseases. Approximately half of the diagnoses (48.3%) were musculoskeletal diseases, 30.1% were skin diseases, and 21.6% were respiratory diseases. Mean age of onset for working in greenhouses was 15.5 years. Percentages of participants with a previous history of working in a different workplace and with a current additional job were 42.6% and 14.2% respectively. Nearly half of participants had workday loss due to those diseases. Almost all participants in the survey were exposed to hazardous chemicals, especially pesticides via routes of inhalation and skin contact as well as ingestion with food and beverages consumed in the workplace. Pesticides were claimed as an occupational risk factor triggering the disease by nearly two-thirds of patients with skin or respiratory system disorders. More than half of all patients and patients with skin diseases or patients with musculoskeletal diseases answered positively to all five questions about work-relatedness of their disease, although the number of the cases referred to authorized university hospital by family physicians was quite few.

A person with an undefined occupational or work-related disease is expected to seek care from a physician in the primary health care services [14]. The nature of work-related or occupational diseases is often not recognized when a patient is consulting a general physician. Lack of awareness and lack of taking a proper occupational history generally are the causes of underreporting. Several studies address this problem. In general, one of the main problems for underdiagnosis of occupational diseases is the lack of proper occupational history and other related information [15]. Although some basic questions on whether symptoms arise at work or whether other colleagues have similar symptoms could be important clues, occupational history is not usually obtained at the expected level, due to intensive work conditions. Several studies address this problem. A study performed in the UK established that 25% of family physicians did not ask about occupation during their examination [16]. In Turkey, a study showed that 43.9% of the physicians who provided outpatient care didn’t ask patients’ occupations [17]. Other problems for underdiagnosis of occupational diseases may include unawareness of notification procedures, need for additional time and effort to complete notification procedures, and unavailability of systems promoting notification [18]. Although notification of occupational diseases is defined by law and reporting is mandatory in Turkey, notification system has some barriers. The occupational or family physicians should direct the patients with occupational disease to the authorized hospitals for the preparation of the files. The file, which is the basis for the recognition of the occupational disease, is sent to SSI which makes the final decision on suspected occupational disease.

In the present study, greenhouse workers with skin, respiratory, and musculoskeletal diseases were evaluated through questions about their work. Although some of the diseases required additional diagnostic procedures (e.g. patch tests for allergic contact dermatitis, serial Peak Expiratory Flow measurements or bronchial challenge tests for asthma), others like low back pain did not, and the algorithm for the diagnosis of occupational diseases began with compatible occupational history [19,20]. By analyzing occupational histories of participants, we obtained clues to diagnose occupational or work-related diseases. Our results also showed that those diseases cause workday loss; thus future studies are needed to evaluate and enhance working conditions of greenhouse workers and to implement specific diagnostic guidelines.

Within the scope of the research, we also obtained data on working life, working environment and working conditions that could affect the health level of greenhouse employees. Although all participants were adults, the mean age of onset for working in greenhouses was 15.5 years. Some participants reported much younger starting ages for greenhouse working, which was part of family business, despite strict prohibitions of current legislation [21]. Agricultural work is one of the most common forms and the most dangerous form of child labor [22]. Most of the participants were business owners or unpaid family workers. In the family business, children begin to work at an early age and spend time with their parents in an indoor greenhouse environment where they are faced with many unfavorable conditions for every age group. Family members can spend the day and even sleep in the greenhouse, especially during the winter months. Moreover, small farmers live where they work, so workplace exposures all too easily migrate into the home. Future studies may be planned to evaluate women’s and children’s health issues related to greenhouse working.

The rates of working in another workplace or having additional work and the frequency of workplace change were high in our study. This phenomenon, which is widespread in the agricultural sector, poses a problem for the implementation and sustainability of occupational health and safety practices and monitoring efforts. Although the average daily working time was within the legal limits on the dates of the research, the workload and the duration of daily working can change according to the seasonal and production stage and can exceed the legal limits. Three-quarters of the participants indicated that they did all kind of work in the greenhouse, although it can be inferred that most of the participants performed all kinds of duties and were exposed to all kinds of effects in the same indoor environment. Lack of statistical significance in the distribution of diseases according to the work may be related to the aforementioned issues.

Participants reported higher exposure frequencies than protective device use. Lack of any statistically significant difference between groups using and not using personal protective equipment in regards to the presence of skin or respiratory disease may directly be related to the characteristics of the equipment used. Two-thirds of the respondents said they used work clothes, but not all were work-specific clothes. Similarly, although two-thirds of participants reported the usage of gloves, the gloves were mostly inappropriate for the task and of poor quality. Less than one-fifth of participants stated that they used masks. It is common practice for farmers in developing countries to apply hazardous pesticides while working barefoot. The level of skin exposure varies with the frequency of pesticide application, the pesticide-active ingredient concentration and whether personal protectors or other protector equipment is being used correctly [3]. In order to prevent the spread of pesticide-related health conditions, precautions such as providing workers with less toxic substances (such as biological control methods [23,24]) adequate protective equipment, training on prevention of pesticide exposures, and implementation of administrative restrictions of working in fields where exposure may occur, ould be taken [3].

Our study’s strengths include the original study design, the large study population size, its impact in raising awareness among stakeholders, and encouragement of further studies. After the research was completed, basic occupational health and safety trainings were held, brochures and banners about occupational diseases were delivered. Thanks to the efforts of the Mersin Public Health Directorate, the frequency of obtaining occupation and work records of family physicians in the region has increased. A workshop demonstrating good practice examples was shared with all stakeholders was also held. Additionally, results of current study will be reinforced by several future intervention studies.

A limitation of the study is that the period of collection of the data, corresponded with both summer months with 38°C in the temperature and the Ramadan month of fasting, so the time period reduced the number of referrals to FHCs compared to other months, despite the absence of any factors causing a selection bias. Additionally, there were no records of occupational health and safety and a lack of information on risk assessments and hazard identification studies and precautionary measures of entry and periodic examinations, as well as family physicians’ workload, duration of loss of capacity due to illness, which may have limited number of referred patients. Other limitations include usage of diagnoses made by family physicians without any further standardized verification, inclusion of participants who were currently at a health care facility far from workplace, lack of standardized evaluation of workplaces, possible increased awareness of participants about the relationship between work and their diseases due to family physicians’ awareness about the study.

To conclude, factors such as younger starting ages of working due to family businesses, frequent job changes due to insecurity, and increased physical risk factors such as heavy lifting, bending, or extreme temperature or chemical risk factors like pesticides and chemical fertilizers, demonstrate that greenhouse work is related to a variety of diseases. Results of our study emphasize the need to objectively evaluate both health conditions and risk factors in greenhouse workplaces and to develop solutions. Our findings are important for awareness raising and initiation of a program for improvement of health and safety in greenhouse workers in the Mersin area, which may be implemented later in the entire greenhouse sector in Turkey.
